# A model for cascading failures with the probability of failure described as a logistic function

**DOI:** 10.1038/s41598-021-04753-z

**Published:** 2022-01-19

**Authors:** Minjung Kim, Jun Soo Kim

**Affiliations:** Department of Chemistry and Nanoscience, Ewha Womans University, Seoul, 03760 Republic of Korea

**Keywords:** Statistical physics, thermodynamics and nonlinear dynamics, Energy grids and networks

## Abstract

In most cascading failure models in networks, overloaded nodes are assumed to fail and are removed from the network. However, this is not always the case due to network mitigation measures. Considering the effects of these mitigating measures, we propose a new cascading failure model that describes the probability that an overloaded node fails as a logistic function. By performing numerical simulations of cascading failures on Barabási and Albert (BA) scale-free networks and a real airport network, we compare the results of our model and the established model describing the probability of failure as a linear function. The simulation results show that the difference in the robustness of the two models depends on the initial load distribution and the redistribution of load. We further investigate the conditions of our new model under which the network exhibits the strongest robustness in terms of the load distribution and the network topology. We find the optimal value for the parameter of the load distribution and demonstrate that the robustness of the network improves as the average degree increases. The results regarding the optimal load distribution are verified by theoretical analysis. This work can be used to develop effective mitigation measures and design networks that are robust to cascading failure phenomena.

## Introduction

Many networks, including infrastructure networks such as electrical power grids, communications systems, and transportation networks, function through strong interactions between components. This interconnectedness suggests that any malfunction of one or several nodes due to random failures or targeted attacks can propagate through the entire system and thus cause system failures. Examples of the widespread impact of these cascading failures include the Western North American blackouts in 1996^1^, collapse of the internet by congestion^2,3^, and systemic risk in financial systems^4–6^. Therefore, given the increasing complexity of the networks that our daily lives largely depend on, the exploration of cascading failures in network context is fundamental to understanding and controlling them. To this end, developing a universal model that can characterize cascading failures in complex networks is essential.

Various approaches have been proposed to describe cascading failures in complex networks^7^, including betweeness centrality model^8–10^, Motter-Lai model^11,12^, and effective efficiency model^13^. In addition, many studies have been conducted focusing on defense strategies against cascading failures^14–16^ and improving the robustness of networks^17–19^. In most cascading failure models, the node of a network is assumed to fail when its load exceeds its capacity. In other words, the probability that a node fails is 0 when the load is smaller than the capacity and 1 when the load is larger than the capacity. If we plot this probability as a function of load, we obtain a Heaviside step function translated by the value of the capacity in the positive load direction. The overloaded node, however, does not necessarily fail and cease to function because most networks have some mitigation measures that allow overloaded nodes to continue functioning. For instance, it has been argued that overloaded power lines do not immediately break down^20^.

**Figure 1:**
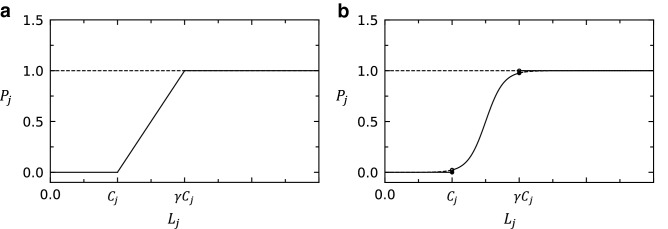
The probability of failure of node *j*, $P_j$ as a function of its load $L_j$ for (**a**) the linear model and (**b**) the logistic model. The value of $P_j$ is 0 when the load of node *j*, $L_j$, is smaller than its capacity $C_j$, and it is 1 when the load $L_j$ exceeds its removal threshold $\gamma C_j$. For $C_j<L_j\le \gamma C_j$, $P_j$ is described as $\frac{L_j-C_j}{\gamma C_j-C_j}$ for the linear model and as the logistic curve in Eq. (4) for the logistic model.

In this direction, a recent study introduced the concept of the removal threshold to model the effects of mitigation measures^21^. Subsequently, a modified model based on the removal threshold has also been proposed^22^. According to the study by Wang et al^21^, the breakdown probability, which is the probability that a node fails (referred to as the probability of failure, hereafter), is as follows: 0 for the range where the load is less than the capacity, 1 for the range where the load is greater than the removal threshold, and between 0 and 1 when the load is greater than the capacity and less than the removal threshold. They further assumed that the probability of failure is linearly proportional to its load when the load is in the range between the capacity and the removal threshold (Fig. 1a). However, the relationship between the probability of failure and the load is likely to be nonlinear rather than linear in most real networks. If the load of a node is slightly larger than its capacity, the mitigation measures generally work well and it is unlikely that the overloaded node would simply collapse. However, as the load increases, the measures become more strained with load handling, and thus the probability of failure increases rapidly. Since the probability cannot be greater than 1, the probability of failure cannot continue to increase as the load increases; the probability must gradually converge to 1. Therefore, in this study, we introduce a logistic function to describe this nonlinear behavior of the probability of failure (Fig. 1b).

Here, using numerical simulations of cascading failures with the probability of failure expressed as a logistic function, we investigate cascading behaviors taking place on scale-free BA networks proposed by Barabási and Albert^23^ and the US airport network^24^. The introduction of the assumption that not all overloaded nodes are removed from the network will increase the robustness of the network; instead the overloaded nodes break down according to the probability of failure. To examine how efficiently our model improves the robustness of the network, we compare our simulation results with those from the simulations of the model with a linear probability of failure^21^ carried out under the same cost. The difference in the robustness of the two models is shown to be highly dependent on the parameter of the initial load distribution and the redistribution of load. In addition, we investigate the optimal value for the parameter of the load distribution where the network is the most robust against cascading failures in our model. The numerical results about the optimal load distribution are verified by theoretical analysis. The topology of the network is a major factor in determining the robustness of the network; thus, the effects of the average degree on the robustness of the network are also investigated.

The rest of the present paper proceeds as follows. In the next section, the cascading failure model with the probability of failure described as a logistic function is introduced. After that, we present our simulation results and analyze those results in terms of the robustness of the network. The optimal load distribution and the topology of the network attaining the most robust network against cascading failures is discussed. We also compare our results with those of the existing model that describes the probability of failure as a linear function. In addition, the simulation results regarding optimal load distribution are verified by theoretical analysis of cascading behaviors. In the last section, we summarize the present work and discuss applications of our findings to improve the robustness of networks.

## Cascading failure model with the probability of failure

Our cascading failure model is defined on a simple undirected and unweighted network. A network consists of nodes and the interconnections between them, called links. For instance, if the network is an electrical power grid, nodes represent generators and links correspond to transmission lines. Since the status of each node is determined by that of its neighboring nodes along the links in a network, the failure of a node can propagate through the entire network by sequentially collapsing the neighboring nodes.

To model cascading failures in a network, two quantities are assigned on each node, i.e., the *load* and the *capacity*. The load on a node is the total amount of work that has to be handled by the node. The capacity represents the maximum load that a node can handle. In our model, we adopt the initial load distribution where the load on node *i* is defined as

1 $$\begin{aligned} L_i \equiv k_i^\alpha , \end{aligned}$$

where $k_i$ is the degree of node *i* and $\alpha >0$ is a tunable parameter that governs the size of the initial load^15,16^. We assume that the capacity of node *i*, $C_i$, is proportional to its initial load, $L_i$^11^, and thus it is expressed as

2 $$\begin{aligned} C_i \equiv \beta L_i, \end{aligned}$$

where $\beta \ge 1$ is a tolerance parameter determining the tolerance of the network against cascading failures.

The simulation for our model goes as follows. At the start of the simulation, we attack and break down one node, triggering a cascading event. Then the load assigned on the collapsed node will be redistributed to its connected nodes along the links. The amount of load that the neighboring node will inherit from the failed node is assumed to be proportional to the initial load of the neighboring node^25–28^. Therefore, if node *i* fails initially, the load transferred to one of its neighboring nodes *j* from node *i* is given by

3 $$\begin{aligned} \Delta L_{ji} \equiv L_i\frac{k_j^\alpha }{\sum _{l \in \Lambda _i}k_l^\alpha }, \end{aligned}$$

where $\Lambda _i$ is the set of nodes directly connected to node *i*.

If the load of node *j* exceeds its capacity by additional load $\Delta L_{ji}$, node *j* is generally considered to collapse and is removed from the network. However, in real networks, the load exceeding the capacity does not necessarily lead to the failure of the node because the network generally has the ability to alleviate the additional load and thus to keep the node functioning. For instance, in traffic networks, when sudden traffic congestion is created, we can take effective measures to ease the traffic, maintaining the function of the node in the traffic network^21^.

As mentioned above, we introduce a logistic function as the probability that a node fails when its load is between its capacity and its removal threshold to model the effect of these mitigation measures. Thus, we write the probability of failure of node *j* as

4 $$\begin{aligned} P_j={\left\{ \begin{array}{ll} 0, &{} L_j\le C_j,\\ \frac{1}{1+e^{-(L_j-\frac{C_j+\gamma C_j}{2})}}, &{} C_j<L_j\le \gamma C_j,\\ 1, &{} L_j> \gamma C_j, \end{array}\right. } \end{aligned}$$

where $\gamma C_j$ is the removal threshold of node *j* ($\gamma \ge 1$). If the load of node *j*, $L_j$, gets larger than $\gamma C_j$, the probability that node *j* fails, $P_j$, becomes 1^21^. This is because the mitigation measures of the network can no longer function when the load is much larger than the capacity. We set this critical value of load at which the probability of failure becomes 1 as the removal threshold.

For $C_j<L_j\le \gamma C_j$, $P_j$ in Eq. (4) represents the logistic function with the $L_j$ value of the sigmoid’s midpoint being $\frac{C_j+\gamma C_j}{2}$ (Fig. 1b). A logistic curve is a type of an S-shaped sigmoid function, whose slope increases from a small value to a maximum value and then decreases again^29^. The logistic curve describes how the probability of failure increases as the load grows. The probability of failure increases as the effectiveness of mitigation measures decreases. When the load is slightly larger than the capacity, the mitigation measures are generally effective, but as the load further increases, the mitigation measures become increasingly strained with handling the load and the probability of failure increases rapidly. After the probability of failure increases significantly, it gradually converges to the maximum value of 1. There are two additional advantages to using the logistic function as the probability of failure. First, its function value is in between 0 and 1, which is one of the axioms of probability^30^. Second, the logistic curve converges to 0 as its argument gets smaller, and to 1 for larger argument. This property is in accordance with our model where the probability of failure is 0 when the load is less than the capacity, and 1 when the load is greater than the removal threshold, as can be seen in Eq. (4).

At every time step of the simulation, the node is considered for removal according to the probability of failure in Eq. (4). The load of the failed node is then redistributed to its connected nodes by the load portioning described in Eq. (3). If the node whose load exceeds its capacity does not fail because the value of the probability of failure is smaller than 1, the load of that node is decreased to its capacity value. The process is repeated until there are no nodes to fail. At the end of the cascading failure trajectory initiated by removing node *i*, we count the total number of failed nodes $S_i$ and divide it by $N-1$ to normalize. We repeat the trajectory by removing each node in a network and obtain *N* normalized number of failed nodes. Then the fragility of the whole network is measured by the order parameter $S_N$:

5 $$\begin{aligned} S_N \equiv \frac{\sum _{i \in V}S_i}{N(N-1)}, \end{aligned}$$

where *V* is the set of nodes in a given network and the summation is over all *i* in *V* such that $1\leqslant i\leqslant N$.

## Results

### Numerical analysis of cascading behaviors

Numerical simulations of cascading failures were performed with the probability of failure described by logistic function to investigate the robustness of the network. In this study, the scale-free BA network by Barabási and Albert^23^ is used as a model network since many natural and man-made systems can be described as scale-free networks^31^. The parameter *m* of the Barabási and Albert model^23^ is set to 3, obtaining the average degree $\langle k \rangle = 2m = 6$. The network size *N*, the total number of nodes in the network, is set to 1000.

**Figure 2:**
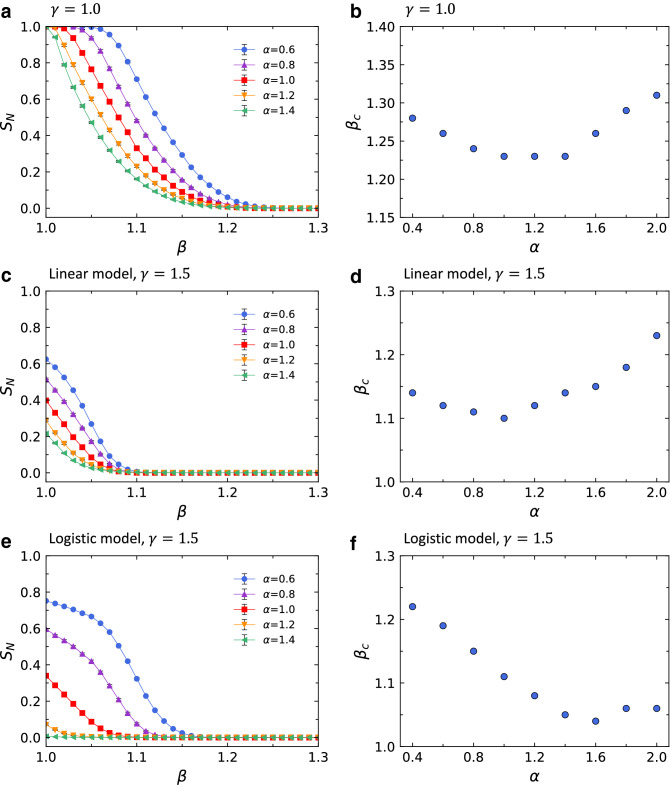
Dependence of the order parameter $S_N$ on the tolerance parameter $\beta$ in Eq. (2) with (**a**) $\gamma =1.0$, (**c**) linear model with $\gamma =1.5$, and (**e**) logistic model with $\gamma =1.5$ and the critical threshold $\beta _c$ as a function of the parameter $\alpha$ in Eq. (1) with (**b**) $\gamma =1.0$, (**d**) linear model with $\gamma =1.5$, and (**f**) logistic model with $\gamma =1.5$. The value of $\gamma =1.5$ is chosen in such a way that the lowest curve of $S_N$ in (**c**) and (**e**) is close to zero at $\beta =1.0$. We use the network size *N*=1000 and the average degree $\langle k\rangle$=6, and each data point is the averaged value for 20 independent runs. Error bars in (**a**), (**c**), and (**e**) are smaller than the symbol size and thus are almost unrecognizable.

To illustrate the effect of the tolerance parameter $\beta$ in Eq. (2) on the robustness of the network, we display $S_N$ as a function of $\beta$ in Fig. 2. We first measure $S_N$ varying the tolerance parameter $\beta$ without introducing the removal threshold ($\gamma =1$ in Eq. (4)) as shown in Fig. 2a. Each data point in Fig. 2a represents an average over 20 independent network realizations. When $\beta$ is close to 1, the probability that cascading failures occur is high because the capacity $C_i$ is similar to the load $L_i$. On the other hand, in the limit of $\beta \rightarrow \infty$, the load of each node $L_i$ cannot be greater than its capacity $C_i$, so there will be no cascades of node failures. Thus, the value of $S_N$ decreases with increasing $\beta$, as shown in Fig. 2a. The parameter $\alpha$ in Eq. (1) also affects the variation of $S_N$. We can see that $S_N$ decreases more quickly as $\alpha$ gets bigger, implying that the way the initial load is assigned on the node and the redistribution of load influence the robustness of the network.

The value of $\gamma$ greater than 1 indicates that mitigation measures are applied to the network, and thus $S_N$ decreases more rapidly when $\gamma =1.5$ than when $\gamma =1.0$ as shown in Figs. 2a,e. To evaluate the efficiency of our cascading failure model in improving the robustness of the network, we compare our logistic probability of failure model (logistic model) with the linear probability of failure model (linear model) proposed in a recent study by Wang et al.^21^. They assume that $P_j$ in Eq. (4) is $\frac{L_j-C_j}{\gamma C_j-C_j}$ for $C_j<L_j\le \gamma C_j$ (Fig. 1a). Previously, Wang et al.^32^ suggested that the cost *w* of preventing cascading failures in a network can be defined as

6 $$\begin{aligned} w=\gamma -1, \end{aligned}$$

where $\gamma$ is the constant in the removal threshold $\gamma C_j$ in Eq. (4). Eq. (6) implies that the cost depends only on the value of $\gamma$. To compare the logistic and linear probabilities of failure under the same cost, we compare them with the same value of $\gamma$. Figures 2c,e display the dependence of $S_N$ on the tolerance parameter $\beta$ in Eq. (2) with the different probabilities of failure, linear and logistic, respectively. It is noted that the results of a comparison between logistic and linear models depend on the parameter $\alpha$ of the load distribution. The linear model gives rise to stronger robustness (i.e., the smaller value of $S_N$) than the logistic model does when $\alpha <1.0$. On the other hand, the logistic model performs better in improving the robustness of networks than the linear model when $\alpha >1.0$. Although we do not show here, for the logistic model with $\gamma \ge 2.5$, values of $S_N$ become almost 0 for all values of $\beta$ when $\alpha \ge 0.6$.

From the $S_N$ vs. $\beta$ curves, we can find that there exists a critical threshold $\beta _c$ dividing the range of $\beta$ into two phases. For $\beta$ larger than $\beta _c$, we do not have any cascading failures. However, once $\beta$ becomes less than $\beta _c$, an initial node failure can trigger a cascading failure. As $\beta _c$ gets smaller, the network is robust over a broader range of the tolerance parameter $\beta$. Accordingly, $\beta _c$ can be used as the measure of the robustness of the network against cascading failures. The value of $\beta _c$ is estimated by finding the point where $S_N$ declined to $0.1\%$. i.e., when the value of $S_N$ becomes 0.001. In Figs. 2b,d,f, we present the dependence of $\beta _c$ on the parameter $\alpha$ for different types of probabilities of failure. Since the smaller $\beta _c$ suggests the stronger robustness of the network, we can see that $\beta _c$ is smaller for $\gamma =1.5$ than for $\gamma =1$ in the figure. In the case of Fig. 2b with $\gamma =1.0$, the network is most robust against cascading failures when $\alpha =1.0$, 1.2, and 1.4. This is consistent with the results of Wang et al^28^ where $\beta _c$ has a minimum value when $\alpha =1.0$. For a linear model with $\gamma =1.5$, the optimal value of $\alpha$ that makes the network the most robust is 1.0 as seen in Fig. 2d. However, for a logistic model with $\gamma =1.5$, the network attains the strongest robustness against cascading failures when $\alpha =1.6$, which can be seen in Fig. 2f. These findings about the optimal value of $\alpha$ in our logistic model will be analyzed theoretically in the following section.

**Figure 3:**
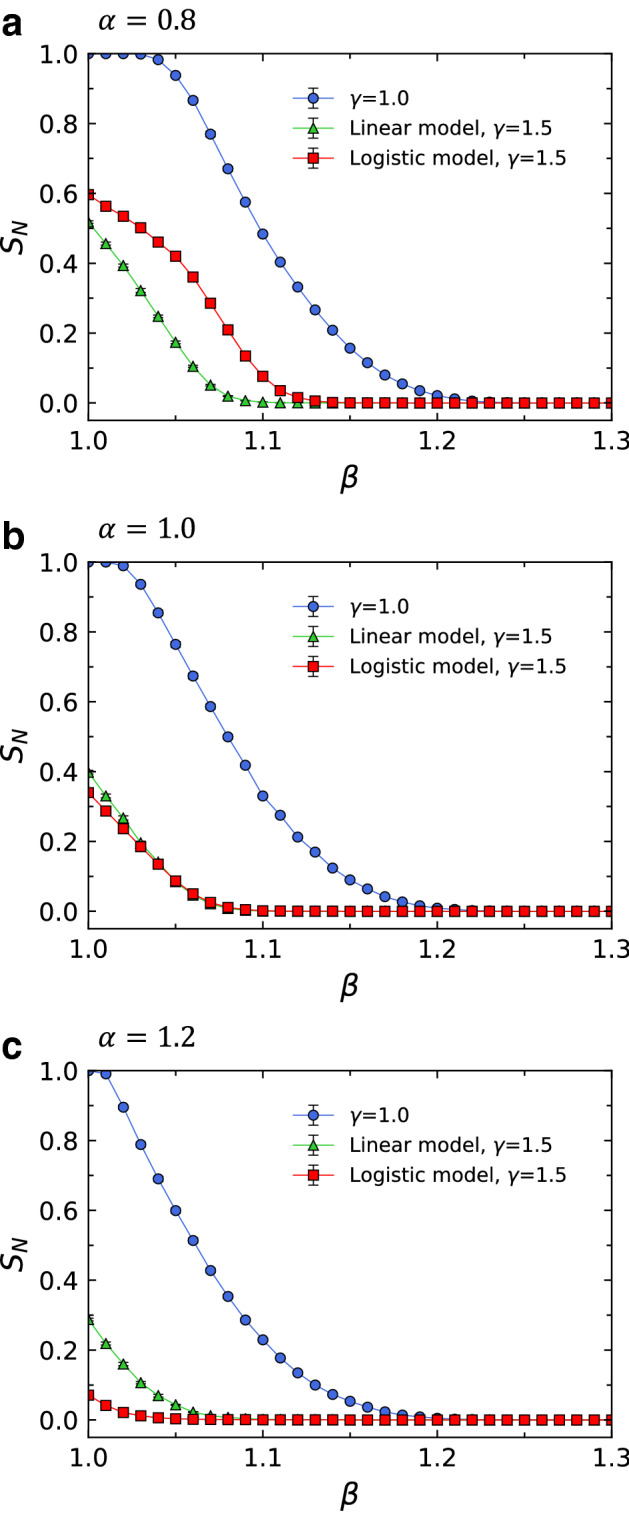
The order parameter $S_N$ as a function of the tolerance parameter $\beta$ in Eq. (2) for different cascading failure models with three different parameters of the load distribution: (**a**) $\alpha =0.8$, (**b**) $\alpha =1.0$, and (**c**) $\alpha =1.2$. The network size *N*=1000 and the average degree $\langle k\rangle$=6 are used, and each data point is obtained by averaging over 20 independent runs.

As can be seen in Figs. 2d,f, for $\alpha >1.0$, the values of $\beta _c$ from the logistic model are smaller than those from the linear model, indicating that the logistic model improves the robustness of networks. However, for $\alpha <1.0$, the values of $\beta _c$ of the logistic model are larger than those of the linear model, suggesting that the linear model enhances the robustness of networks. For $\alpha =1.0$, the logistic and linear models have similar $\beta _c$ values. These results suggests that the logistic model is an effective means of mitigating cascading failures only when the values of $\alpha >1.0$. The curves in Figs. 2c,e also show that the performance comparison results of the logistic and linear models are highly dependent on the load distribution. However, the difference between the values of $S_N$ of the two models at each value of $\alpha$ is not clearly shown in the figure. Therefore, in Fig. 3, we present $S_N$ vs. $\beta$ curves for two models at three different parameters of the load distribution with $\alpha =0.8$, $\alpha =1.0$, and $\alpha =1.2$. Fig. 3 reveals that the performance of the linear model is better than the logistic model for $\alpha <1.0$ (Fig. 3a) whereas the logistic model is more effective at mitigating cascading failures than the linear model for $\alpha >1.0$ (Fig. 3c). For $\alpha =1.0$, logistic and linear models have similar $S_N$ values, indicating that they have the same level of ability to prevent cascading failures (Fig. 3b).

**Figure 4:**
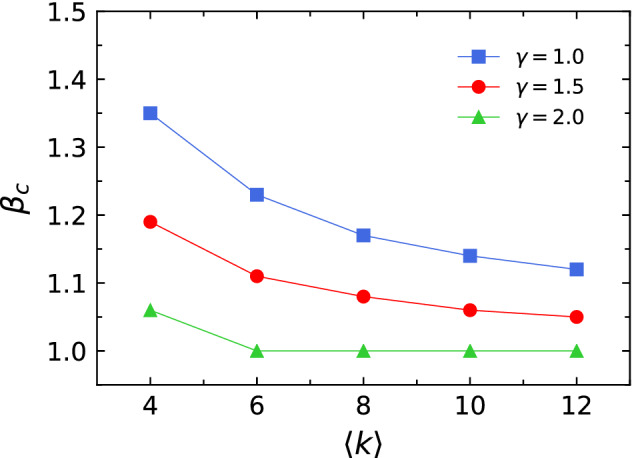
The tolerance parameter $\beta _c$ as a function of the average degree $\langle k\rangle$ for $\gamma =1.0$, $\gamma =1.5$, and $\gamma =2.0$. The parameter $\alpha$ in Eq. (1) is set to 1.0 and $\beta _c$ is obtained from the averaged $S_N$ *vs*. $\beta$ curve for 20 independent runs.

To examine the effect of network topology on the robustness of networks in our logistic model, we also ran the simulations in scale-free networks with diverse average degrees. In Fig. 4, we present $\beta _c$ when $\alpha =1$ as a function of the average degree $\langle k \rangle$ at three different levels of mitigation measures with $\gamma =1.0$, $\gamma =1.5$, and $\gamma =2.0$. The value of $\alpha$ was fixed at 1 to exclude the influence of the load distribution. Figure 4 clearly reveals that the critical threshold $\beta _c$ and the average degree $\langle k \rangle$ are inversely proportional to each other for all values of $\gamma$. For $\gamma =2.0$, $\beta _c$ decreases and then becomes constant at 1.0 as $\langle k \rangle$ gets larger because the minimum value of $\beta _c$ is 1.0. The value of $\beta _c$ also has a negative correlation with the value of $\gamma$, indicating that the bigger the value of $\gamma$, the more robust the network.

**Figure 5:**
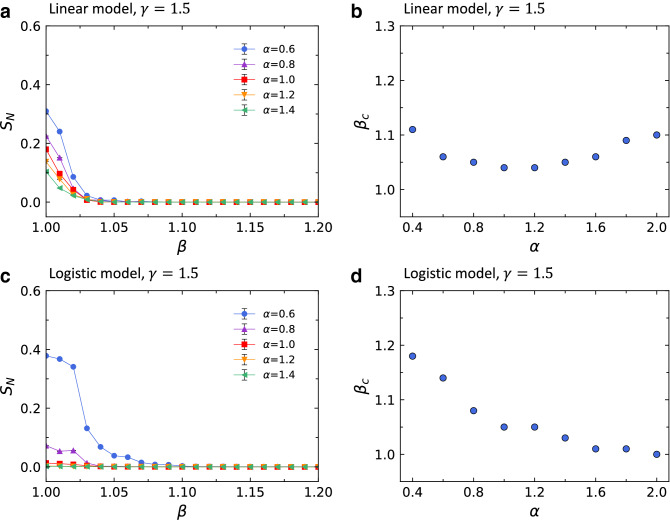
The robustness of airport network based on the linear and the logistic models. Dependence of the order parameter $S_N$ on the tolerance parameter $\beta$ in Eq. (2) with (**a**) linear model with $\gamma =1.5$, and (**c**) logistic model with $\gamma =1.5$ and the critical threshold $\beta _c$ as a function of the parameter $\alpha$ in Eq. (1) with (**b**) linear model with $\gamma =1.5$, and (**d**) logistic model with $\gamma =1.5$ are displayed.

To apply our model to real networks, we also simulated cascading failures in the US airport network as of 1997 where nodes represent airports, and a link connects between two airports when there is a direct flight between them ^24^. Figure 5 presents $S_N$ as a function of $\beta$ and the dependence of the critical threshold $\beta _c$ on the parameter $\alpha$. In Figs. 5b,d, we can see that the data points for the linear model lie lower than those for the logistic model for $\alpha \le 1.2$, while the behavior is reversed for $\alpha >1.2$. These results suggest that the logistic model is more efficient than the linear model for large values of $\alpha$, while the linear model is a better mitigation measure for small values of $\alpha$, consistent with the results based on BA network presented in Fig. 2. However, a difference was observed in the value of $\alpha$ at which the behavior of $\beta _c$ is reversed between BA network and the US airport network: $\alpha =1.0$ for BA network and $\alpha =1.2$ for the US airport network.

### Theoretical analysis of cascading behaviors

To validate the numerical results from the previous section, we examine our cascading failure model theoretically. Our purpose of theoretical analysis is to find the value of $\alpha$ in Eq. (1) when $\beta _c$ is the minimum. To this end, we consider the condition where the cascading process initiated by removing node *i* is terminated. The conditions that the neighboring node *j* of node *i* should satisfy for $\gamma =1$ and $\gamma >1$ are given by

7 $$\begin{aligned} {\left\{ \begin{array}{ll} L_j+\Delta L_{ji}<C_j, &{} \gamma =1,\\ \frac{1}{1+e^{-(L_j+\Delta L_{ji}-\frac{C_j+\gamma C_j}{2})}}<p, &{} \gamma >1,\\ \end{array}\right. } \end{aligned}$$

where *p* is the random number between 0 and 1. If we substitute Eq. (1) for $L_j$ and Eq. (3) for $\Delta L_{ji}$ into Eq. (7), we obtain

8 $$\begin{aligned} {\left\{ \begin{array}{ll} k_j^\alpha +k_i^\alpha \frac{k_j^\alpha }{\sum _{l \in \Lambda _i}k_l^\alpha }<\beta k_j^\alpha , &{} \gamma =1,\\ \frac{1}{1+e^{-(k_j^\alpha +k_i^\alpha \frac{k_j^\alpha }{\sum _{l \in \Lambda _i}k_l^\alpha }-\frac{(1+\gamma ) \beta k_j^\alpha }{2})}}<p, &{} \gamma >1.\\ \end{array}\right. } \end{aligned}$$

The conditions of Eq. (8) can be rewritten in a simpler form as

9 $$\begin{aligned} {\left\{ \begin{array}{ll} 1+\frac{k_i^\alpha }{\sum _{l \in \Lambda _i}k_l^\alpha }<\beta , &{} \gamma =1,\\ \frac{2}{1+\gamma }(1+\frac{k_i^\alpha }{\sum _{l \in \Lambda _i}k_l^\alpha }+\frac{ln(\frac{1}{p}-1)}{k_j^\alpha })<\beta , &{} \gamma >1.\\ \end{array}\right. } \end{aligned}$$

Here we approximate $\sum _{l \in \Lambda _i}k_l^\alpha$ in Eq. (9) by its expectation value as

10 $$\begin{aligned} \sum _{l \in \Lambda _i}k_l^\alpha \simeq E(\sum _{l \in \Lambda _i}k_l^\alpha )=\sum _{k'=k_{\mathrm{min}}}^{k_{\mathrm{max}}}k_iP(k'|k_i)k'^ \alpha , \end{aligned}$$

where $P(k'|k_i)$ is the conditional probability that node *i* with the degree $k_i$ is directly connected to the node with the degree $k'$, and $k_{\mathrm{min}}$ and $k_{\mathrm{max}}$ are the minimum and maximum degrees of nodes in a network, respectively. There is no degree-degree correlation in BA networks, so we have $P(k'|k_i)=\frac{k'P(k')}{\langle k \rangle }$. Thus, Eq. (10) can be expressed as

11 $$\begin{aligned} \sum _{k'=k_{\mathrm{min}}}^{k_{\mathrm{max}}}k_iP(k'|k_i)k'^ \alpha =\frac{k_i}{\langle k \rangle }\sum _{k'=k_{\mathrm{min}}}^{k_{\mathrm{max}}}P(k')k'^ {\alpha +1}=\frac{k_i\langle k^{\alpha +1} \rangle }{\langle k \rangle }. \end{aligned}$$

Based on Eqs. (10) and (11), the inequalities of Eq. (9) are given as

12 $$\begin{aligned} {\left\{ \begin{array}{ll} 1+\frac{k_i^{\alpha -1}\langle k \rangle }{\langle k^{\alpha +1} \rangle }<\beta , &{} \gamma =1,\\ \frac{2}{1+\gamma }(1+\frac{k_i^{\alpha -1}\langle k \rangle }{\langle k^{\alpha +1} \rangle }+\frac{ln(\frac{1}{p}-1)}{k_j^\alpha })<\beta , &{} \gamma >1.\\ \end{array}\right. } \end{aligned}$$

From Eq. (12), we can see that the critical threshold $\beta _c$ depends on $\alpha$ and *p*, thus, we calculate $\beta _c$ in three ranges of $\alpha <1$, $\alpha =1$, and $\alpha >1$ and two ranges of $p>\frac{1}{2}$ and $p<\frac{1}{2}$ as

13 $$\begin{aligned} \beta _c={\left\{ \begin{array}{ll} {\left\{ \begin{array}{ll} 1+\frac{k_{\mathrm{min}}^{\alpha -1}\langle k \rangle }{\langle k^{\alpha +1} \rangle }, &{} \alpha<1,\\ 1+\frac{\langle k \rangle }{\langle k^2 \rangle }, &{} \alpha =1,\\ 1+\frac{k_{\mathrm{max}}^{\alpha -1}\langle k \rangle }{\langle k^{\alpha +1} \rangle }, &{} \alpha>1,\\ \end{array}\right. } &{} \gamma =1,\\ {\left\{ \begin{array}{ll} {\left\{ \begin{array}{ll} \frac{2}{1+\gamma }(1+\frac{k_{\mathrm{min}}^{\alpha -1}\langle k \rangle }{\langle k^{\alpha +1} \rangle }+\frac{ln(\frac{1}{p}-1)}{k_{\mathrm{max}}^\alpha }), &{} \alpha<1,\\ \frac{2}{1+\gamma }(1+\frac{\langle k \rangle }{\langle k^2 \rangle }+\frac{ln(\frac{1}{p}-1)}{k_{\mathrm{max}}}), &{} \alpha =1,\\ \frac{2}{1+\gamma }(1+\frac{k_{\mathrm{max}}^{\alpha -1}\langle k \rangle }{\langle k^{\alpha +1} \rangle }+\frac{ln(\frac{1}{p}-1)}{k_{\mathrm{max}}^\alpha }), &{} \alpha>1,\\ \end{array}\right. } &{} p>\frac{1}{2},\\ {\left\{ \begin{array}{ll} \frac{2}{1+\gamma }(1+\frac{k_{\mathrm{min}}^{\alpha -1}\langle k \rangle }{\langle k^{\alpha +1} \rangle }+\frac{ln(\frac{1}{p}-1)}{k_{\mathrm{min}}^\alpha }), &{} \alpha<1,\\ \frac{2}{1+\gamma }(1+\frac{\langle k \rangle }{\langle k^2 \rangle }+\frac{ln(\frac{1}{p}-1)}{k_{\mathrm{min}}}), &{} \alpha =1,\\ \frac{2}{1+\gamma }(1+\frac{k_{\mathrm{max}}^{\alpha -1}\langle k \rangle }{\langle k^{\alpha +1} \rangle }+\frac{ln(\frac{1}{p}-1)}{k_{\mathrm{min}}^\alpha }), &{} \alpha>1.\\ \end{array}\right. } &{} p<\frac{1}{2},\\ \end{array}\right. } &{} \gamma >1,\\ \end{array}\right. } \end{aligned}$$

Here we make another approximation that sets the random number *p* in Eq. (13) to its expectation value. Since *p* is the random number between 0 and 1, its expectation value is 0.5. Replacing *p* in Eq. (13) by 0.5, we can obtain

14 $$\begin{aligned} \beta _c={\left\{ \begin{array}{ll} {\left\{ \begin{array}{ll} 1+\frac{k_{\mathrm{min}}^{\alpha -1}\langle k \rangle }{\langle k^{\alpha +1} \rangle }, &{} \alpha<1,\\ 1+\frac{\langle k \rangle }{\langle k^2 \rangle }, &{} \alpha =1,\\ 1+\frac{k_{\mathrm{max}}^{\alpha -1}\langle k \rangle }{\langle k^{\alpha +1} \rangle }, &{} \alpha>1,\\ \end{array}\right. } &{} \gamma =1,\\ {\left\{ \begin{array}{ll} \frac{2}{1+\gamma }(1+\frac{k_{\mathrm{min}}^{\alpha -1}\langle k \rangle }{\langle k^{\alpha +1} \rangle }), &{} \alpha <1,\\ \frac{2}{1+\gamma }(1+\frac{\langle k \rangle }{\langle k^2 \rangle }), &{} \alpha =1,\\ \frac{2}{1+\gamma }(1+\frac{k_{\mathrm{max}}^{\alpha -1}\langle k \rangle }{\langle k^{\alpha +1} \rangle }), &{} \alpha>1,\\ \end{array}\right. }&\gamma >1. \end{array}\right. } \end{aligned}$$

We can see that there is a difference only in the constant factor between the two cases, $\gamma =1$ and $\gamma >1$ in Eq. (14).

To find the value of $\alpha$ when $\beta _c$ is the minimum, we compare $\frac{k_{\mathrm{min}}^{\alpha -1}\langle k \rangle }{\langle k^{\alpha +1} \rangle }$ in the case of $\alpha <1$ and $\frac{\langle k \rangle }{\langle k^2 \rangle }$ in the case of $\alpha =1$ for $\gamma =1$, $\gamma >1$ in Eq. (14) as

15 $$\begin{aligned} \frac{k_{\mathrm{min}}^{\alpha -1}\langle k \rangle }{\langle k^{\alpha +1} \rangle }=\frac{k_{\mathrm{min}}^{\alpha -1}\langle k \rangle }{\frac{1}{N}{\sum \limits _{i=1}^{i=N} k_i^{\alpha +1}}}=\frac{\langle k \rangle }{\frac{1}{N}{\sum \limits _{i=1}^{i=N} k_i^2({\frac{k_i}{k_{\mathrm{min}}}})^{\alpha -1}}}>\frac{\langle k \rangle }{\frac{1}{N}{\sum \limits _{i=1}^{i=N} k_i^2}}=\frac{\langle k \rangle }{\langle k^2 \rangle }. \end{aligned}$$

Hence, we have $\beta _c(\alpha =1)<\beta _c(\alpha <1)$ from Eq. (14) and the inequality of Eq. (15). The comparison between $\frac{k_{\mathrm{max}}^{\alpha -1}\langle k \rangle }{\langle k^{\alpha +1} \rangle }$ in the case of $\alpha >1$ and $\frac{\langle k \rangle }{\langle k^2 \rangle }$ in the case of $\alpha =1$ for $\gamma =1$, $\gamma >1$ in Eq. (14) can be made in a similar way as

16 $$\begin{aligned} \frac{k_{\mathrm{max}}^{\alpha -1}\langle k \rangle }{\langle k^{\alpha +1} \rangle }=\frac{k_{\mathrm{max}}^{\alpha -1}\langle k \rangle }{\frac{1}{N}{\sum \limits _{i=1}^{i=N} k_i^{\alpha +1}}} =\frac{\langle k \rangle }{\frac{1}{N}{\sum \limits _{i=1}^{i=N} k_i^2({\frac{k_i}{k_{\mathrm{max}}}})^{\alpha -1}}}>\frac{\langle k \rangle }{\frac{1}{N}{\sum \limits _{i=1}^{i=N} k_i^2}}=\frac{\langle k \rangle }{\langle k^2 \rangle }. \end{aligned}$$

This inequality along with Eq. (14) indicates that $\beta _c(\alpha =1)<\beta _c(\alpha >1)$. Combining these two results, $\beta _c(\alpha =1)<\beta _c(\alpha <1)$ and $\beta _c(\alpha =1)<\beta _c(\alpha >1)$, we can conclude that $\beta _c$ has a minimum value when $\alpha =1$ for $\gamma =1$, $\gamma >1$. This analytical results are in good agreement with the simulations results for $\gamma =1$ as can be seen in Fig. 2b. For $\gamma >1$, however, the theoretical prediction deviates slightly from the simulation results. For $\gamma =1.5$, a network reaches the strongest level of robustness when $\alpha =1.6$ as shown in Fig. 2f. The reason for this deviation is that we approximated $\sum _{l \in \Lambda _i}k_l^\alpha$ and the random number *p* by their expectation values, $\frac{k_i\langle k^{\alpha +1} \rangle }{\langle k \rangle }$ and 0.5, respectively.

## Discussion

We have presented a new cascading failure model by modeling the probability of failure of an overloaded node as a logistic function. The probability of failure is adopted to consider the effects of mitigation measures of the network. Then we focus on the nonlinear relationship between the probability of failure and the load on the node and introduce a logistic function to characterize it. We have performed numerical simulations of cascading failures on BA networks and a real airport network to investigate the cascading behaviors of our model.

The proposed probability of failure improves the robustness of the network compared to the case where the probability of failure is not adopted as expected. To assess the efficiency of our cascading failure model in improving the robustness of the network, we compare the results of our logistic model with those of the linear model. We have found that the comparison results of the robustness using the two models depend on the way the load is initially assigned on the node and redistributed between nodes. The conditions that enhance a network’s robustness were also examined regarding the load distribution and the topology of the network. The optimal value for the parameter of the load distribution has been found, and is also investigated by theoretical analysis. In terms of the topology of the network, the network becomes more robust as the average degree of the network increases.

The main objective of our study is to introduce a new cascading failure model and to investigate its behavior and efficiency in improving the robustness of the network. We used BA network as a model system since BA networks can represent many natural and artificial systems. Therefore, the results of this study can be used to analyze cascading failure events in real systems. The main findings of our study is that the results of comparison between logistic and linear probabilities of failure depend on the initial load distribution and the redistribution of load. This can be used to devise effective anti-impact strategies against cascading failures in complex networks.
